# Detection of Post‐ Hematopoietic Stem Cell Transplant Bronchiolitis Obliterans Syndrome (BOS)—The Use of Airway Oscillometry

**DOI:** 10.1002/ppul.71831

**Published:** 2026-09-16

**Authors:** Samuel B. Goldfarb, Richard Cooper, Jane Koo, Kasiani C. Myers, Laura Walkup, Jason Woods, Eleanor Cook, Brian Hils, Adam Lane, Julian Allen, Stella M. Davies, Christopher Towe

**Affiliations:** ^1^ Departments of Pediatrics, Division of Pediatric Pulmonary and Sleep Medicine University of Minnesota Minneapolis Minnesota USA; ^2^ Department of Pediatrics, Division of Bone Marrow Transplantation and Immune Deficiency, Cincinnati Children's Hospital Medical Center University of Cincinnati Cincinnati Ohio USA; ^3^ Center for Pulmonary Imaging Research Cincinnati Children's Hospital Medical Center Cincinnati Ohio USA; ^4^ Minderoo Children's Comprehensive Cancer Centre Sydney Children's Hospital Sydney Australia; ^5^ School of Clinical Medicine University of NSW Sydney Australia; ^6^ Department of Pediatrics, Division of Pulmonary and Sleep Medicine, Children's Hospital of Philadelphia University of Pennsylvania Philadelphia Pennsylvania USA; ^7^ Department of Pediatrics, Division of Pulmonary Medicine, Cincinnati Children's Hospital Medical Center University of Cincinnati Cincinnati Ohio USA

## Abstract

**Background:**

Bronchiolitis obliterans syndrome (BOS) is a devastating complication of allogeneic hematopoietic stem cell transplantation (HSCT) characterized by onset of obstructive lung disease. Oscillometry is a novel pulmonary function assessment technology which superimposes pressure waves on normal tidal breathing, and the alterations in flow and pressure caused by the external waves are measured. Oscillometry assesses physiological parameters that provide insight into respiratory mechanics. We hypothesized that oscillometry may allow for detection of small airway disease to diagnose BOS, especially in children unable to perform spirometry.

**Methods:**

We conducted a cross‐sectional study to characterize oscillometry findings in patients with BOS compared to a control cohort of HSCT patients without BOS. PFT testing were performed on all patients at the time of oscillometry testing.

**Results:**

Thirty‐six patients post HSCT were approached, 18 with BOS and 18 controls. These two groups were similar in demographic parameters. BOS patients had significantly abnormal spirometry values. Oscillometry results demonstrated significant differences between the two cohorts in X5, AX, R5, R5‐19 and (R5‐19)/R5. There were significant differences between BOS and transplant control groups when assessing the association between oscillometry parameter and spirometry parameters.

**Conclusion:**

In our cohort oscillometry revealed significant differences between the BOS and non‐ BOS cohort. Resistance at R5 Hz and the difference between R5‐R19 Hz, which characterizes peripheral lung resistance, were both abnormally increased when compared with non‐ BOS subjects. In addition, reactance parameters, X5 and AX, which correlate with lung stiffness demonstrated significant differences between the BOS and non‐ BOS cohorts. Our data reveal a strong correlation between oscillometric and spirometric abnormalities in patients with established BOS originally identified by standard spirometry testing. In addition, oscillometry suggests localization of increased resistance in the lung periphery supporting our hypothesis that oscillometry might be complementary to spirometry, and enhance our ability to diagnose BOS.

**Trial Registration:** ClinicalTrials.gov identifier: NCT04098445

## 1 Introduction

Hematopoietic stem cell transplantation is performed to treat many disorders, including hematological malignancy, immune deficiency, hemoglobinopathy, or inborn errors of metabolism. Bronchiolitis obliterans syndrome (BOS) is a devastating complication of allogeneic hematopoietic stem cell transplantation (HSCT) characterized by new onset of an obstructive lung defect. BOS diagnostic criteria are outlined in the NIH consensus criteria that rely on lung function testing, specifically spirometry, which assesses forced expiratory flow volumes. NIH criteria for BOS include a decline in spirometry measurements of FEV1, and FEV1/FVC ratio with or without air trapping on chest CT and absence of infection [1]. Spirometry requires forced expiratory maneuvers that can be technically difficult for young and/or ill children [2, 3, 4, 5, 6]. New diagnostic criteria for BOS in pediatric patients account for limitations in performing pulmonary function testing (PFT) by spirometry in pediatrics in this often sick population. The ability to diagnose BOS in pediatrics based on current NIH guidelines has been challenged, in part due to lack of sensitivity in this age group. The new NIH BOS pediatric criteria use relative change in lung function as a diagnostic criterion [7]. Patients with BOS are commonly asymptomatic early in the disease course, highlighting the importance of effective routine screening [8]. Current practice in pediatrics has been changed to include alternative testing modalities, including multiple breath washout to measure lung clearance index and CT chest imaging. These new guidelines did not include oscillometry as at the time of publication as there were not enough data to support its use [7, 9].

Oscillometry is a novel pulmonary function assessment technology which superimposes pressure waves on normal tidal breathing. Oscillometry assesses physiological parameters that provide insight into respiratory mechanics. Importantly, oscillometry is not effort dependent. Oscillometry measures respiratory system impedance which consists of two components, resistance (R_rs_) and reactance (X_rs_). Oscillometry measures these two components at various frequencies and provides information related to airway size and tissue resistive and elastic properties. R increases with airway narrowing. Resistance at 5 Hz (R5) is proposed to reflect total (central and peripheral) airway and tissue resistance, while R19 better reflects central resistance because the longer time constants of peripheral airways may cause those airways to incompletely fill at higher frequencies. Therefore R5–R19 is thought to represent resistance of the peripheral airway. We can further infer that (R5‐19)/R5 represents the proportion of total resistance in the periphery. Reactance at 5 Hz (X5) reflects respiratory system elastic recoil and elastance (1/compliance), and is a marker of respiratory system stiffness, whereas at higher frequencies it reflects inertial properties. Furthermore, respiratory stiffness will be greater in settings of small airway parallel pathway time constant inhomogeneities that cause incomplete filling of peripheral lung units. We report reactance at 5 Hz (X5), where elastic reactance predominates. Area of reactance (AX) (the area between the *x*‐axis and the reactance curve from the lowest frequency measured to the resonant frequency) also reflects the elastic properties of the lung, as well as inhomogeneity of small airway obstruction and restrictive lung disease. Lastly, the resonant frequency (fres) is the frequency at which the elastic and inertial forces of the respiratory system are equal. It increases with increased parenchymal and airway stiffness, and also with younger age, since smaller lung volumes have an absolutely lower compliance.

Oscillometry has been successfully performed on children as young as 3 years of age [10]. Cho et al. have shown that oscillometry may be more sensitive than spirometry at detecting peripheral airway dysfunction post lung transplant [11]. In studies of pediatric asthma patients, the difference between resistance at 5 and 19 Hz (R5–R19) and AX (area under the reactance curve) have demonstrated abnormal values when correlated with abnormal spirometry values in the detection of obstructive lung disease [12, 13]. Correlation of spirometry values and oscillometry has also been reported in lung transplant recipients with chronic lung allograft dysfunction (CLAD) and in patients with post infectious bronchiolitis obliterans (PIBO) [14, 15].

The National Heart Lung and Blood Institute (NHLBI), the National Institute of Child Health and Human Development (NICHD) and the National Cancer Institute (NCI) convened a workshop of multi‐disciplinary experts to discuss pulmonary complications after HSCT in children in April 2018. The executive summary of the meeting identified an urgent need for novel diagnostic and therapeutic strategies to identify, prevent and treat lung injury [16]. We hypothesized that oscillometry may aid in the detection of small airway disease to diagnose BOS.

## 2 Methods

**Figure 1:**
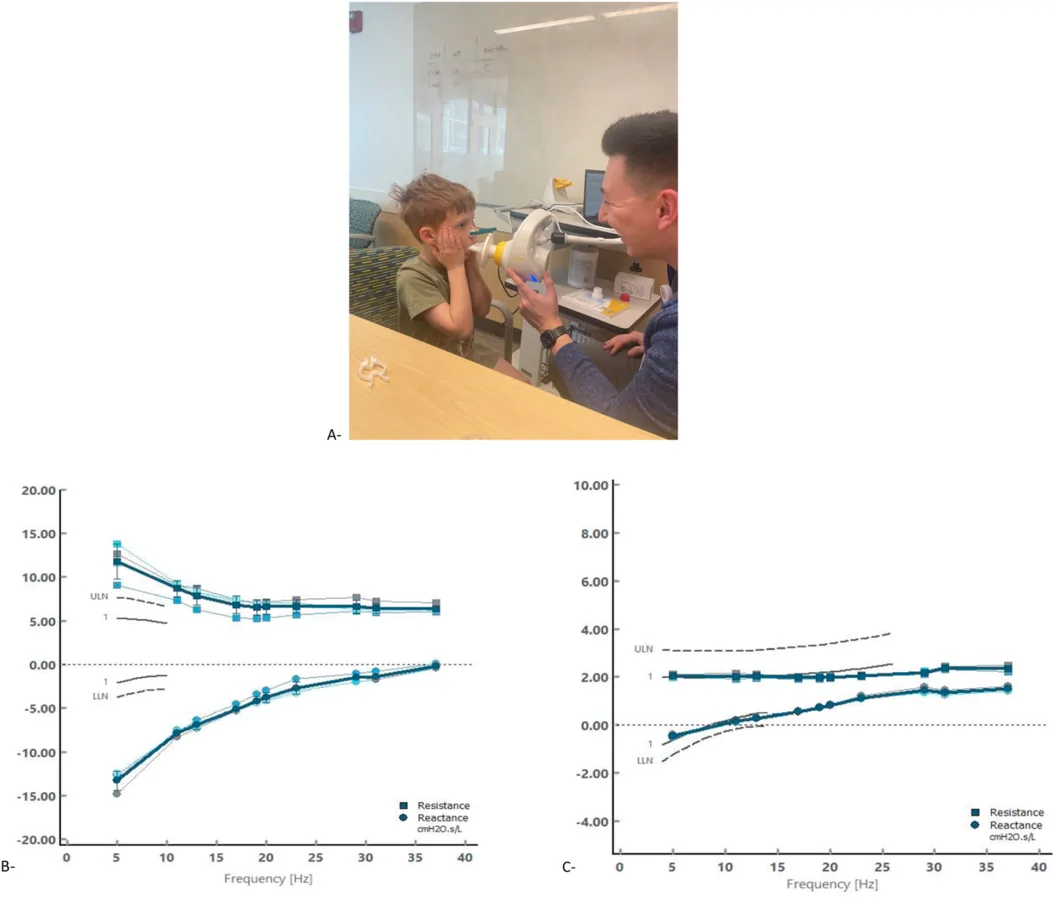
(A) Healthy child performs portable airway oscillometry. The child sits upright with mouth over the mouthpiece. Nose is occluded and cheeks supported. Child performs tidal breathing for 20–30 s (duration of testing is age dependent) for a minimum of three tests. (B) Oscillometry results in patient with BOS: 12yo female, 7 years post HSCT for CML. C‐ Oscillometry results in control patient without BOS: 23yo male, 2 years post HSCT for immunodeficiency. Solid lines labeled “1” are the predicted values. Dashed lines ULN and LLN represent the upper limit of normal resistance and the lower limit of normal reactance, respectively. [Color figure can be viewed at wileyonlinelibrary.com]

We performed portable airway oscillometry in children and young adults with diagnosed BOS at Cincinnati Children's Hospital Medical Center (CCHMC) per NIH consensus criteria and new ATS consensus criteria [1, 9] and post‐HSCT controls without BOS. BOS was generally diagnosed within 2 years post HSCT [17, 18, 19]. The study was approved by the CCHMC institutional review board (Study Identification Number CCHMC‐2023‐0024) and all patients and/or guardians signed informed consent. Controls were all patients at our site that were an average of at least 2 years post HSCT without the clinical diagnosis of BOS and had oscillometry testing performed using the same oscillometry device as BOS subjects. Control subjects were selected consecutively during the timeframe of enrollment of BOS patients from April 2023–August 2024. Control cohort post study follow up to assess for development of cGVHD or BOS concluded July 1, 2026. All subjects were tested using a similar sinusoidal airwave oscillometer (Tremoflo C100, THORASYS, Montreal, CA), encompassing a range of prime frequencies between 5 and 37 Hz. This portable device was brought to the patients’ clinic room during routine follow up. Testing requires the patient to perform tidal breathing through a mouthpiece for 20–60 s (age dependent), for a minimum of three tests (Figure 1A). Nose clips were applied to avoid nasal leak, and cheeks were compressed to avoid upper airway shunt compliance. PFT testing was performed according to ATS/ERS guidelines and center protocol [20]. PFT testing and oscillometry testing were performed on the same day. When both tests were performed, oscillometry testing always preceded spirometry testing, according to ATS/ERS guidelines. Since oscillometry results are height dependent, oscillometry variables were adjusted for height in a series of linear models.

## 3 Statistical Analysis

Categorical and continuous data are described by frequency (%) and median (IQR), respectively. Normative data set z scores were used to assess the association between BOS status and the oscillometry variables as well as to assess associations between oscillometry and spirometry. Global lung initiative (GLI) data set was used to determine spirometry *z* scores. Values were compared to Ducharme and Oostveen normative values based on age. Oscillometry *z*‐scores were directly converted to published *z* scores [21, 22]. Differences in *z*‐scores between groups were assessed with two‐sided Wilcoxon rank sum test. Spearman correlation coefficient (*r*) and its *p*‐value are given for the association between two continuous variables. All analysis were conducted in R version 4.5.1. Statistical significance is set a *p* = 0.05.

## 4 Results

### 4.1 Patients and Spirometry Results

Thirty‐six patients were approached, 18 with BOS and 18 controls with no known pulmonary abnormality. All patients had undergone a HSCT. One patient (aged 12yo) was unable to perform the test due to sclerotic GVHD affecting the ability to form an effective seal around the mouthpiece. Table 1 shows patient demographics. Median age was 16 years, and median time from HSCT for the BOS cohort was 4.5 years (0.9–15.5 years) and for the control cohort 2.0 years (0.6–13.8. years). The most common primary diagnosis was malignancy (17/36), followed by bone marrow failure and primary immunodeficiency (10/36 and 6/36 respectively). The BOS patient cohort had significantly abnormal PFTs as expected, compared to our control population who had received an HSCT with no history of pulmonary cGVHD (Table 2). No patients in the control cohort developed BOS after a minimum follow up of 2 years post testing. One subject in the control cohort did develop non‐pulmonary cGVHD.

**Table 1:** Patient demographics.

	All patients	BOS patients	Post‐HSCT Controls
	(*n* = 36)	(*n* = 18)	(*n* = 18)
Demographics			
Median time post HSCT		4.5 years (0.9–15.5)	2.0 years (0.6–13.8)
Median age (years)	16 (7–25)	17 (10–25)	15 (7–23)
Gender	16 Females (44%)	10 Females (55%)	6 Females (33%)
Median height (cm)	154 (114–184)	156 (126–172)	150 (114–184)
Primary diagnosis			
Malignancy	17 (47%)	12 (67%)	5 (28%)
Bone marrow failure	10 (28%)	1 (6%)	9 (50%)
Immunodeficiency	6 (17%)	3 (17%)	3 (17%)
Benign hematology	2 (6%)	2 (11%)	0
Genetic/Metabolic	1 (3%)	0	1 (6%)

**Table 2:** Spirometry variables in patients post HSCT in BOS and Non‐BOS cohorts.

Spirometry values	Total	BOS cohort	Non‐BOS cohort	*p* value
FVC *z* score	32	−2.2 (−2.8 to −1.2)	−0.2 (−0.9 to 0.7)	< 0.001
FEV1 *z* score	32	−4.4 (−5.2 to −3.5)	−0.3 (−1.3 to 0.5)	< 0.001
FEV1/FVC *z* score	33	−3.3 (−3.8 to −3.0)	−0.8 (−1.1 to 0.4)	< 0.001
FEF25%–75% *z* score	31	−4.7 (−5.3 to −4.3)	−0.7 (−1.1 to −0.1)	< 0.001

*Note:* Spirometry *z* scores from GLI race neutral normative data. FVC‐forced vital capacity, FEV1‐forced expiratory volume at 1 s, FEF25%–75%‐ forced expiratory flow between 25% and 75% of forced vital capacity, BOS‐Bronchiolitis obliterans syndrome.

### 4.2 Oscillometry Results

**Figure 2:**
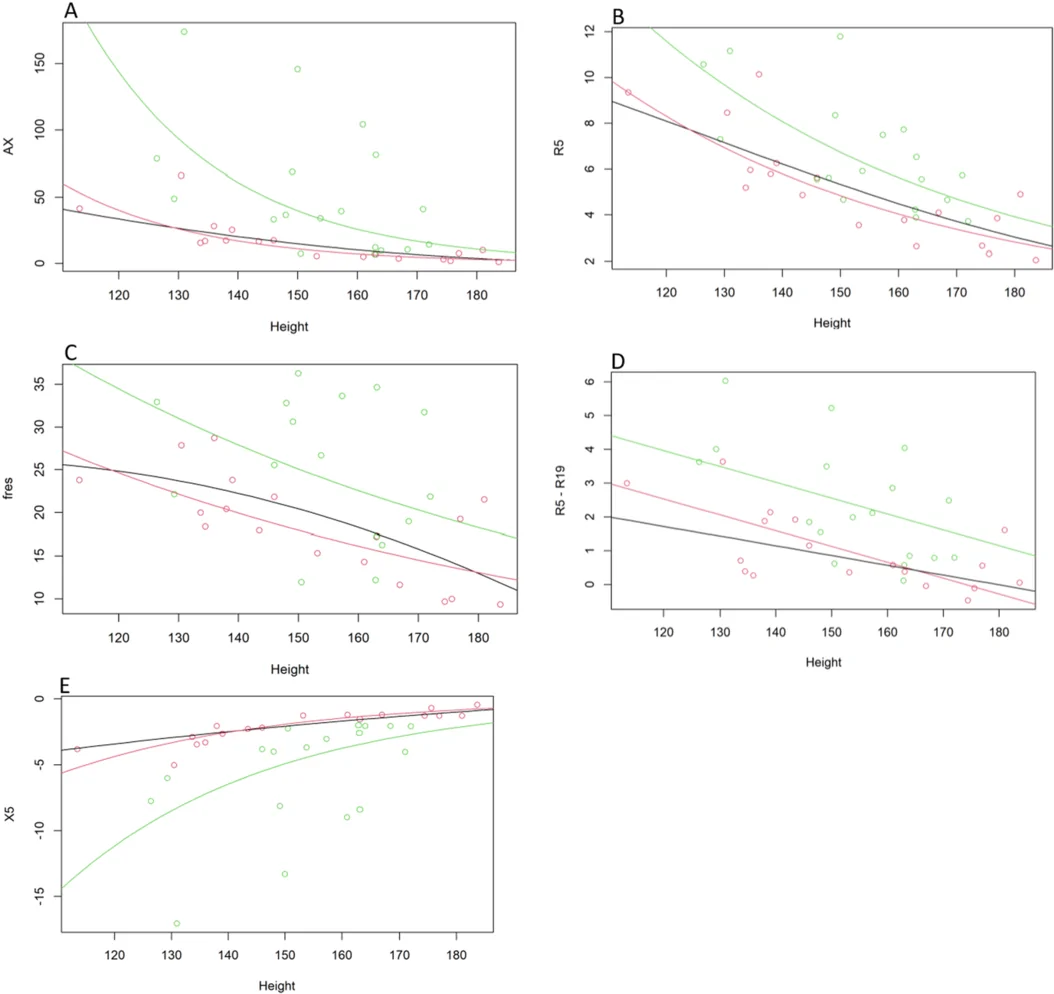
(A–E) representing AX (2A), R5 (2B), fres (2C), R5‐R19 (2D) and X5 (2E) plotted for height in regression equations for Control (red) and BOS (green) Patients. Ducharme reference equation represented by the black line. Height in centimeters. [Color figure can be viewed at wileyonlinelibrary.com]

Oscillometry results differed markedly between the two cohorts. Oscillometry values and corresponding z‐scores are reported in Table 3. This comparison demonstrated significant differences between the two cohorts in X5, fres, AX, R5, R5‐19, and (R5‐19)/R5, the latter being a derived measure of proportion of airway resistance residing in the small airways (see Discussion) (Table 3). Characteristic oscillometry patterns of obstructive lung disease are shown in Figure 1B,C. An example of a BOS subject (Figure 1B) compared to a control subject (1C) shows increased (more negative) reactance X5, increased resistance, R5, a higher fres, and increased area under the curve, from R5 to fres, AX. Figure 2A–E plot oscillometry values with height.

**Table 3:** Oscillometry outcomes in HSCT controls and BOS subjects.

Variable	BOS median (IQR)	Control median (IQR)	*p*	BOS *Z* score	Control *z* score range?	*p*
X5 cmH2O/L/s	−3.9 (−8.0 to −2.3)	−1.8 (−2.8 to −1.3)	< 0.001	−2.9 (−8.7 to −2.0)	−0.0 (−0.5 to −2.0)	< 0.001
fres, Hz	26.1 (18.5 to 32.8)	18.8 (14.6 to 21.7)	0.004	1.1 (−0.3 to 2.4)	−0.5 (−0.8 to 0.3)	0.062
AX cm/L	37.8 (12.7 to 76.0	12.8 (5.1 to 17.3)	< 0.001	1.8 (0.6 to 3.5	−0.4 (−0.7 to 0.3)	< 0.001
R5 cmH2O/L/s	5.8 (4.9 to 7.7	4.9 (3.6 to 5.9)	0.001	1.1 (0.1 to 2.4)	0.5 (−1.0 to 0.3)	0.002
R5‐19 cmH2O/L/s	2.0 (0.8 to 3.6)	0.6 (0.3 to 1.8)	0.003	1.4 (0.4 to 2.8)	0.2 (−0.9 to 0.6)	0.003
(R5‐19)/R5, %	33.4 (17.9–42.9)	14.5 (3.6–32.8)	0.011	N/A	N/A	N/A

*Note:* Oscillometry values in BOS Subjects versus controls. Values compared using Ducharme and Oostveen reference value *Z* scores based on patient age.

Abbrevation: BOS, bronchiolitis obliterans syndrome.

In a multivariable linear model that included malignancy status (Y/N) and BOS (Y/N), BOS was still significantly associated with X5, fres, AX, R5, R5‐19, and (R5‐19)/R5. Malignancy was not found to be significantly associated with any OSC variable in these analyzes.

### 4.3 Oscillometry Findings Correlate With Spirometry

**Figure 3:**
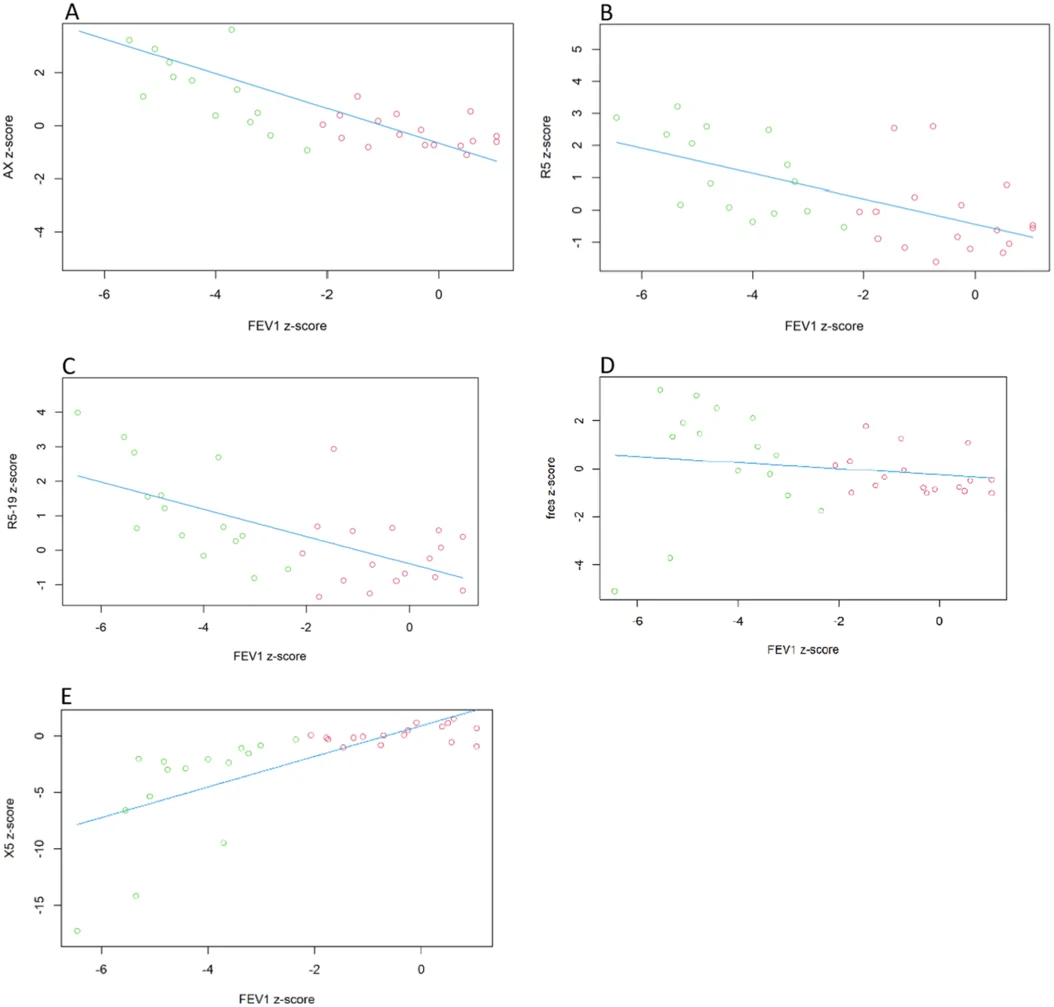
A‐E ‐ AX (3A), R5 (3B), R5‐19 (3C), (3D) fres and (3E) X5 are *z*‐score values plotted vs FEV1 *z* score values with Control (red) and BOS (green). [Color figure can be viewed at wileyonlinelibrary.com]

The association between spirometry and oscillometry variables reflective of airway obstruction and airway reactance (R5, R5‐19, AX, X5 and fres) are summarized in Table 4. Of note variables, X5, AX, R5 and R5‐19, were all statistically significantly correlated with FVC, FEV1, FEV1/FVC and FEF25%–75% *z*‐scores. Comparison of spirometry *z* score values and oscillometry *z* score values revealed a strong correlation. Spirometry, FEV1, and oscillometry variables are illustrated in Figure 3A‐E.

**Table 4:** Relationship between Spirometry and Oscillometry measurements (*Z*‐scores): Analysis compared FEV1, FEV1/FVC, FVC and FEF25‐75 Z‐scores per GLI and oscillometry parameter X5, AX, R5 and R5‐R19 Z‐scores, using Ducharme and Oostveen reference values.

		FEV1		FEV1/FVC		FVC		FEF25‐75%	
Oscillometry value	*N*	*r*‐value	*p*	*r*‐value	*p*	*r*‐value	*p*	*r*‐value	*p*
X5, cmH2O/L/s	36	0.71	< 0.001	0.60	< 0.001	0.77	< 0.001	0.88	< 0.001
AX, cm H2O/L	36	−0.77	< 0.001	−0.53	0.002	−0.74	< 0.001	−0.74	< 0.001
R5, cmH2O/L/s	36	−0.62	< 0.001	−0.65	< 0.001	−0.52	0.002	−0.65	< 0.001
R5‐R19cmH2O/L/s	36	‐0.63	< 0.001	−0.55	0.014	−0.56	0.001	−0.55	0.001

## 5 Discussion

Oscillometry has the potential to improve the timeliness of diagnosis of BOS after HSCT by being easier to use in small children, and perhaps by increased sensitivity compared with spirometry. We tested oscillometry in HSCT patients divided into two equal cohorts, one of patients who developed chronic graft versus host disease (cGVHD) of the lung (BOS), and one of patients without BOS. Patients in the BOS cohort had abnormal spirometry values with a significant obstructive pattern, including a decline in FEV1 and FEV1/FVC consistent with diagnostic criteria for BOS [1, 7, 9]. We sought to identify similar significant differences in oscillometry parameters to characterize the significant obstructive lung disease known to occur in BOS. We found statistically significant differences between spirometric and oscillometric variables as well as significant correlation between spirometry, and reactance and resistance of the respiratory system. Resistance at 5 Hz (R5) and the difference between R5‐R19 Hz, which characterizes peripheral lung resistance, were both abnormally increased when compared with non‐ BOS subjects. Similar findings have been demonstrated in pediatric patients with other obstructive lung diseases such as asthma, cystic fibrosis, and patients with bronchiolitis obliterans from either HSCT, lung transplant or post‐infectious bronchiolitis obliterans (PIBO) [3, 5, 10, 15, 23, 24, 25].

Reactance parameters, X5 and AX, correlate with lung stiffness and our study demonstrated significant differences between the BOS and non‐BOS cohorts. These findings are similar to those in other forms of peripheral obstructive lung disease [10, 15, 26, 27, 28] This is because with heterogeneous small airway disease, the varying time constants between parallel peripheral diseased airways mean that some distal lung units incompletely fill during high frequency oscillatory flow, leading to a restrictive‐like profile. This was elegantly described by Otis et al as frequency dependence of compliance [29]. Furthermore, spirometry may be insensitive to small airway obstruction. Oscillometry can potentially improve this sensitivity by partitioning between central and peripheral airway obstruction. Since lower frequency resistance, for example, R5 is thought to represent resistance of the entire tracheobronchial, whereas higher frequency resistance, for example, R19 is thought to be more representative of central airways, R5‐R19 has been taken to represent peripheral airway resistance. We have therefore taken (R5‐R19)/R5, a previously described oscillometry outcome [30], to represent the proportion of total resistance residing in the peripheral airways. In normal adult subjects, most of the total resistance resides in the central airways because the total cross‐sectional area is smallest there [31]. In this study, we have shown that the percent of resistance residing in the small airways was 2.0/5.8 in BOS subjects, and 0.6/4.9 in controls (Table 3). In other words, 33% of total resistance was in the small airways in BOS subjects, but only 15% of total resistance was in the small airways in controls. These results are consistent with the teaching that 80%–90% of resistance in normal subjects lies in the central airways and only 10%–20% in the periphery [32], which has therefore been referred to as “the quiet zone” [33]. Our data show this distribution is markedly different in BOS subjects. This is a novel mechanistic finding that spirometry is less able to localize. Thus, oscillometry may allow us to better “hear” that “quiet zone” than spirometry.

Findings consistent with both restrictive and obstructive lung disease have been validated in other cohorts and specifically in lung transplant recipients where chronic allograft dysfunction has two forms, restrictive and obstructive [24]. Patients with bronchiolitis obliterans organizing pneumonia (BOOP) have pathology that involves both small airways and pulmonary parenchyma [34]. This can lead to increases in both airway and tissue resistance and reactance. In addition, many of the therapies these patients receive before their transplants can cause changes in both pulmonary and chest wall impedance [35, 36, 37].

Partitioning respiratory system resistance and elastance between airway and tissue components would require equipment and techniques more sophisticated than that used in this study, and is discussed in detail by Bates et al [38] and King et al. [4] Since the pathology of BOS involves both components, these more sophisticated techniques will likely yield further important mechanistic insights in the future.

There are other methods to assess BOS in patients post HSCT. Westrupp et al. have demonstrated the feasibility of nitrogen multiple breath washout (MBW) for screening children post‐HSCT in a small prospective cohort (*n* = 3 patients with BOS) in which they demonstrated an elevated lung clearance index at 97.5% washout. (LCI_2.5_). MBW differs from oscillometry, and in younger children or sick patients may be difficult to perform; however, Westrupp et al. and others have shown MBW to be more technically successful compared to spirometry [39]. Similar findings were found in patients with Cystic Fibrosis [40]. Hardaker et. al. assessed early peripheral airway disease in a small cohort of HSCT patients, reporting changes in LCI values and in AX and fres in forced oscillometry variables [41].

Our data reveal a strong correlation between oscillometry and spirometric abnormalities in patients with established BOS originally identified by standard spirometry testing supporting our hypothesis that oscillometry might enhance our ability to diagnose BOS. Similar studies have been performed in adult in lung transplant recipients that develop BOS and confirm the correlation between declining spirometry values [14, 24, 42]. Neither study was able to determine if oscillometry provided a pathway to early diagnosis [14]. There is only one other reported study comparing oscillometry and spirometry in pediatric HSCT patients with BOS. Lee et al report assessed impulse oscillometry in pediatric patients, including 20 with BOS after HSCT. Comparison of oscillometry variables with spirometry in patients with BOS showed similar findings to our study with strong correlation between individual oscillometry variables and individual spirometry values [25].

Our study has limitations and strengths. One limitation is that neither oscillometry nor spirometry measure absolute lung volumes, which can only be accomplished by plethysmography in patients with obstructive disease. A second limitation is the Lack of plethysmography and maximal/minimal inspiratory pressure measurements which limited our ability to preclude restrictive components due to chest wall abnormalities and respiratory muscle weakness. Future studies are warranted to better describe this patient population with expanded lung function testing. Another limitation is that our study design, a cross‐sectional study with a relatively small sample size, unmatched cohorts with potential selection bias did not lend itself to determining whether oscillometry is more or less sensitive than spirometry in the diagnosis of BOS. Furthermore, the design of the study did allow which lung function testing could help diagnose BOS earlier. Since the subjects were divided into BOS and non‐BOS‐ groups using spirometric criteria, spirometry was diagnostic of BOS by definition. Strengths include our application of an innovative pulmonary function testing strategy to a cohort of post‐HSCT children and young adults. We have defined the unique oscillometry characteristics of BOS. This technique is portable, not effort dependent and relatively inexpensive. Oscillometry can be performed by younger children than spirometry. Another strength is that by providing a measure that delineates the proportion of respiratory system resistance that resides in the peripheral airways, we suggest mechanistic insight into the major site of airway resistance in BOS. This is an important signal which warrants further study, in particular longitudinal screening of a large cohort of HSCT recipients to determine if oscillometry can identify onset of BOS earlier than conventional strategies.

## Author Contributions

**Samuel B. Goldfarb:** conceptualization, writing – original draft, methodology, validation, writing – review and editing, data curation, supervision, investigation. **Richard Cooper:** data curation, validation, investigation. **Jane Koo:** investigation, conceptualization, writing – review and editing, formal analysis, validation. **Kasiani C. Myers:** conceptualization, investigation, funding acquisition, writing – original draft, methodology, validation, writing – review and editing, formal analysis. **Laura Walkup:** conceptualization, investigation, writing – review and editing, methodology, formal analysis. **Jason Woods:** conceptualization, investigation, writing – review and editing, methodology, formal analysis, supervision, funding acquisition. **Eleanor Cook:** conceptualization, investigation, writing – original draft, methodology. **Brian Hils:** investigation, validation, data curation. **Adam Lane:** conceptualization, writing – review and editing, validation, methodology, formal analysis, data curation. **Julian Allen:** conceptualization, writing – review and editing, validation, methodology, formal analysis. **Stella M. Davies:** conceptualization, investigation, funding acquisition, writing – review and editing, validation, methodology, supervision. **Christopher Towe:** project administration, supervision, data curation, formal analysis, writing – review and editing, methodology, validation, writing – original draft, funding acquisition, investigation, conceptualization.

## Conflicts of Interest

The authors declare no conflicts of interest.

## Data Availability

The data that support the findings of this study are available on request from the corresponding author. The data are not publicly available due to privacy or ethical restrictions.
